# Use of Reflectance Measurements for the Detection of N, P, K, ADF and NDF Contents in Sainfoin Pasture

**DOI:** 10.3390/s8117275

**Published:** 2008-11-14

**Authors:** Sebahattin Albayrak

**Affiliations:** Suleyman Demirel University, Faculty of Agriculture, Department of Field Crops, Isparta-Turkey

**Keywords:** Sainfoin pasture, spectroradiometer, reflectance, quality variables, nitrogen

## Abstract

The objective of this study was to determine the relationships between nitrogen (N), phosphorus (P), potassium (P), acid detergent fiber (ADF) and neutral detergent fiber (NDF) contents of sainfoin (*Onobrychis sativa* Lam.) pasture and canopy reflectance. Canopy reflectance measurements were made by using a portable spectroradiometer. An experiment was conducted in the Turkey in May and June in 2007 and 2008. Sainfoin pasture N, P, K, ADF and NDF contents correlated linearly with the reflectance ratios R780/650 (0.61≤ r^2^ ≤0.80) and first derivatives of the reflectance ratios 760/630 (0.70≤ r^2^ ≤0.84). Linear equations between each forage variable and reflectance or first derivatives reflectance had high r^2^ (0.68≤ r^2^ ≤0.83 and 0.79≤ r^2^ ≤0.90, respectively) in R780 and R760 wavelengths. In stepwise regression of the reflectance (in 460, 550, 650 and 780 nm wavelengths), the r^2^ of predicted and measured N, P, K, ADF and NDF contents of sainfoin pasture were (0.85, 0.85, 0.78, 0.81 and 0.74, respectively), in stepwise regression of the first derivatives of reflectance (in 440, 530, 630 and 760 nm wavelengths), the r^2^ of predicted and measured N, P, K, ADF and NDF contents of sainfoin pasture were (0.87, 0.91, 0.83, 0.93 and 0.86, respectively). Our results suggest that canopy reflectance in blue, green, red and near infrared wavebands with NIR/Red and NDVI ratios can be used for nondestructive prediction of forage quality variables in sainfoin pasture.

## 1. Introduction

Remote sensing techniques, based on measuring the reflected radiation from plant canopies, have the potential of evaluating the nutrient contents of many plants within the field of view of the sensor [1]. Multispectral reflectance measured with hand-held radiometers has been used to estimate many plant parameters of interest [2]. Recently, researchers have evaluated remote sensing techniques for estimating the nutrient contents of growing crops by determining the appropriate wavelength or combination of wavelengths to characterize crop nutrient deficiencies [3]. In the visible spectrum (400-700 nm), leaf reflectance is low because of absorption by photosynthetic pigments (mainly chlorophylls and carotenoids). In the near-infrared spectrum (700-900 nm), where there are no strong absorption features, the magnitude of reflectance is governed by structural discontinuities encountered in the leaf [4]. A relationship between spectral reflectance, particularly visible absorption and macronutrients such as phosphorous, potassium, magnesium and calcium is expected due to their effect on the photosynthetic process in plants [5, 6]. For example, phosphorous is fundamental to tissue composition as well as being one of the components of the nucleic acids and enzymes. Potassium is also important, both for activating enzymes responsible for the metabolism of carbohydrates and in the apical dominance [7, 8]. These elements are therefore responsible for both the photosynthetic process and the tissue composition of plants, and hence related to the visible absorption bands [9]. Nitrogen exhibits specific absorption features in the shortwave infrared [10] and is also responsible for the metabolic function of the chlorophyll [8]. Everitt *et al.* [11] investigated relationships between leaf reflectance and leaf nitrogen or chlorophyll concentration in buffelgrass and concluded that leaf reflectance at 500 and 550 nm highly correlated with leaf N and chlorophyll concentrations. More recently, Lamb *et al.* [12] reported that leaf reflectance in red-edge range of wavelengths (690-740 nm) could be used to estimate leaf N concentration and total N content of ryegrass. Mutanga *et al.* [8] found that concentrations of N, P, K, Ca and Mg in grass pastures could be predicted using continuum-removed absorption features of measurements of canopy reflectance. Starks *et al.* [13] found that forage N, NDF, and ADF concentrations closely and linearly correlated with pasture canopy reflectance. If the forage quality variables and biomass production can be predicted from nondestructive and timely measurements of canopy reflectance in a few wavebands via a spectroradiometer, it would further reduce laborious field sampling and sample processing procedures [14]. Visible near infrared (VNIR) spectroradiometer has been developed and applied to estimation of chemical composition in plant. Measurement of sample by spectroradiometer is rapid, nondestructive, chemical reagents are not necessary. Most of the published studies show that VNIR can accurately estimate the content of several organic components in field crops. However, similar studies on estimation of forage quality parameters using VNIR spectroradiomer are limited.

In present study, some remote sensing indices may be used to rapidly estimate N, P, K, ADF and NDF of sainfoin pasture over the growing season. The objective of this study was to determine the relationships between forage quality variables of sainfoin pasture and spectral reflectance.

## 2. Material and Methods

### 2.1. Experimental Location

This study was conducted during the 2007 and 2008 growing seasons at Isparta (37° 45′ N, 30° 33′ E, elevation 1,035 m), located in the Mediterranean region of Turkey. The field of study was a 5 ha nongrazed, sainfoin pasture, established in 2006. The major soil characteristics, based on the method described by Rowell [15] were found to be as follows: the soil texture was clay; organic matter, total salt, lime were 1.2%, 0.2% and 7%, respectively. Sulphur, extractable P and exchangeable K were 11 mg kg^-1^, 3.2 mg kg^-1^ and 114 mg kg^-1^, respectively, and pH was 7.1.

### 2.2. Measurements

Canopy reflectance measurements were collected from 325 to 1,150 nm (1 nm intervals) with a portable spectroradiometer (Analytical Spectral Devices Inc.; Boulder, CO, USA). The optical sensor of the spectroradiometer was mounted on a boom 1.5 m above and perpendicular to the soil surface. The radiometer had 10° field of view, producing a view area with a 35 cm diameter. A Spectralon (Labshere, Inc.; North Sutton, NH, USA) reference panel (white reference) was used to optimize the ASD (Analytical Spectral Devices Inc.; Boulder, CO, USA) instruments for taking canopy reflectance measurements at each sampling area. The canopy reflectance data were expressed as relative values by dividing them by the white reference panel reflectance readings.

Reflectance measurements were conducted on a clear and cloudless day between 10:00 and 12:00 local time on 10 May, 17 May, 24 May, 31 May and 7 June of the experiment year 2007 and on 8 May, 15 May, 22 May, 29 May and 5 June of the 2008 experiment year. Reflectance measurements were taken at five adjacent points at each sampling date, so a total of 50 sainfoin pasture measurements were made in 2007 and 2008. Reflectance was measured before flowering stage throughout full flowering stage in the sainfoin pasture.

All sainfoin vegetation in a 625 cm² area within the ASD field of view was clipped within 1 cm of the ground surface after canopy reflectance measurements. Sainfoin samples were immediately dried, weighed, and ground for determinations of ADF (Acid detergent fiber) and NDF (Neutral detergent fiber) concentrations according to standard laboratory procedures of forage quality analysis outlined by Ankom Technology (http://www.ankom.com/00_products/product_a2000.shtml; verified 13 September 2008). N (Nitrogen) content was calculated by Kjeldahl method [16]; K (Potassium) content of samples was determined using an atomic spectrophotometer after digesting the samples with HClO_4_:HNO_3_ (1:4) [17]; P (phosphorus) content was determined by vanadomolibdophosphoric yellow colour method [16].

### 2.3. Data Analysis

The reflectance values measured by VNIR (Visible near infrared) spectroradiometer was recorded via ASD ViewSpect® software (Nik System Inc.; CO, İstanbul, TR) as log 1/R (R=Reflectance). The reflectance data were combined into four broad wavebands; blue (450-520 nm), green (520-600 nm), red (630-690 nm) and near infrared (NIR, 760-900 nm). Coefficients of determination (r^2^) between the forage variables and canopy reflectance values in each broad waveband were calculated using the REG procedure in SAS [18]. The highest reflectance was determined in near infrared waveband. A reflectance value at this waveband was separately recorded for each forage quality parameters. The simple ratio of reflectance was defined as near infrared (NIR) to red regions (R(NIR)/R(red)). The normalized difference vegetation index (NDVI) was defined as (R(NIR) - R(red))/(R(NIR) + R(red)) [2]. Regression analysis applied to forage N, P, K, ADF and NDF contents between reflectance and first derivatives of reflectance values and laboratory analysis. First derivatives of reflectance were calculated according to Lamb *et al.* [12]. Predicted values for N, P, K, ADF and NDF contents were obtained by using regression equations. Additionally, each measured forage quality parameter was treated as a response variable in a stepwise regression [18] to determine relationships between the N, P, K, ADF and NDF availability and reflectance or first derivatives of the reflectance in four most important wavebands. In conclusion, r^2^ values were estimated as a result of the stepwise regression analysis between measured and predicted values.

## 3. Results and Discussion

### 3.1. Nutritive Value

Nitrogen ranged between 2.15 and 3.54%, phosphorus was between 0.16 and 0.94%, potassium was between 1.03 and 2.88%, ADF was between 27.54 and 37.76% and NDF was between 35.43 and 44.21% in the two growing seasons (Table 1). Overall, P and K had the greatest (CV 44.21-31.05%) and N, ADF and NDF had smallest (CV 5.56-14.64%) variability among these forage variables. These results are consistent with findings in ungrazed forage fields, where the CV of forage yield and P and K concentration were greater than the CV of ADF and NDF among sainfoin genotypes, harvest dates, and years [19, 20]. Wide ranges in forage quality parameters provided the ideal data sets for development and validation of reflectance in these forage quality variables [21].

### 3.2. Correlation of Forage Quality with Broadband Reflectance

**Figure 1.**
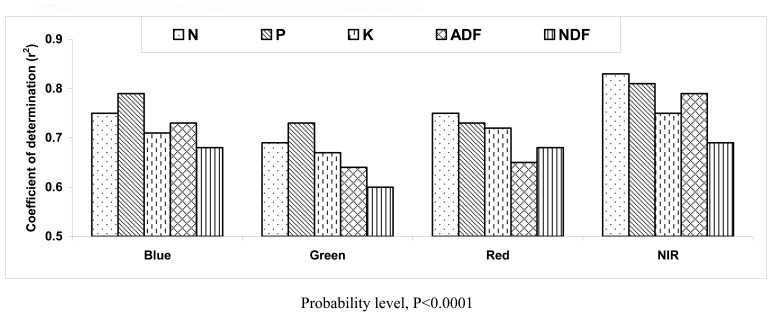
Coefficients of determination (r^2^) of forage nitrogen (N), phosphorus (P), potassium (K), acid detergent fiber (ADF) and neutral detergent fiber (NDF) with canopy reflectance in broad wavebands of blue (450-520 nm), green (520-600 nm), red (630-690 nm) and near infrared (NIR, 760-900 nm) (n=50).

Among the four broad wavebands, reflectances in blue, green, red and NIR bands were most highly correlated with forage N, P, K, ADF and NDF, CP concentration (Figure 1). Most r^2^ values of these forage quality variables with canopy reflectance in a single broadband were statistically significant (P <0.0001) because of a large data set (n=50). Using the reflectance in any broad waveband could explain 69 to 83% of the variation in N, 73 to 81% in P, 67 to 75% in K, 64 to 79% in ADF, and 60 to 69% in NDF availability. These results indicate that correlations of standing pasture canopy reflectance in single broad wavebands with measured forage quality variables of sainfoin are high (Figure 1). Therefore, broad waveband reflectance and other data analysis methods were investigated to determine if relationships between pasture canopy reflectance and forage quality could be improved to more accurately predict forage productivity and nutritive values using remotely sensed data [21].

### 3.3. Relationships between Simple Ratio of Reflectance and Forage Quality Variables

The simple ratio of reflectance in near infrared (NIR) to red regions (R(NIR)/R(red)) of the electromagnetic spectrum and the normalized difference vegetation index (NDVI), defined as (R(NIR) - R(red))/(R(NIR) + R(red)), are the most widely used indices in precision agricultural production [22, 23]. The R(NIR)/R(red) and NDVI are mainly used for predicting plant canopy coverage, leaf area index and herbage mass, and for detecting plant biotic and abiotic stresses [24]. NIR/Red and NDVI with the concentrations of N, P, K, ADF and NDF in the sainfoin pasture are presented in Tables 2 and 3. The NIR/RED explained 61-80% of the variation in most forage nutritive value parameters. The first derivatives of NIR/RED explained 82, 80, 84, 76 and 70% of the variance, respectively, in forage N, P, K, ADF and NDF. The NDVI explained 72-80% of the variation in most forage nutritive value parameters. The first derivatives of NDVI explained 82, 82, 76, 79 and 77% of the variance, respectively, in forage N, P, K, ADF and NDF. Aase and Tanaka [25] reported a relationship between green leaf dry matter and NIR/red ratios, and suggested that reflectance measurements could be used to estimate leaf dry matter or leaf area measurements in spring and wheat (*Triticum aestivum* L.). Stone *et al.* [26] demonstrated that total plant N could be estimated using spectral radiance measurements at the red (671 nm) and NIR (780 nm) wavelengths. Starks *et al.* [24] found that ratios of canopy reflectance in near infrared to red (NIR/RED) waveband was highly correlated with concentration of CP in herbage but the relationships between reflectance ratios and ADF and NDF concentrations of herbage were low. Present study, it was found that relationships between reflectance ratios and N,P,K, ADF and NDF concentrations of sainfoin pasture herbage were high (Table 2, 3). In general, canopy reflectance depends not only on leaf morphological and biochemical characteristics of species [1, 8], but also on the degree of vegetation canopy closure because exposed soils directly affect canopy reflectance features [14]. It should be noted that, in the present study, vegetation canopy of the sainfoin pasture was closed. Thus, any influence due to soil reflectance was minimized. Therefore, when extending our findings to other forage species or to more open canopies of legume pastures, soil effects on plant canopy reflectance must be taken into account.

### 3.4. Relationships between Broadband Reflectance and Forage Quality Variables

The r^2^ values of N, P, K, ADF and NDF with reflectance and first derivatives of reflectance were high in most wavebands (Figure 1). Although reflectance values in blue, green and red wavebands were significantly correlated with these forage quality variables, reflectance near infrared waveband (780 nm) had the greatest r^2^ with N, P, K, ADF and NDF. Park *et al.* [27] reported that live green vegetation has low reflectance in the visible red portion of the spectrum because of absorption by leaf pigments, and high reflectance in the near-infrared (NIR) part of the spectrum owing to scattering in the cellular structure of the leaf mesophyll. Reflectance monotonically increases from the visible to the NIR for dead brown vegetation. Similarly, Xu *et al.* [28] found that in the visible spectrum (400-700 nm), leaf reflectance is low because of absorption by photosynthetic pigments (mainly chlorophylls and carotenoids). In the near-infrared (NIR) (750-900 nm), where there are no strong absorption features, the magnitude of reflectance is governed by structural discontinuities encountered in the leaf. These results are consistent with the present results.

Linear equations and r^2^ values, derived from the spectral data in near infrared waveband, are given in Table 4. The reflectance explained 83, 81, 75, 79 and 68% of the variance, respectively, in forage N, P, K, ADF and NDF. The first derivatives of reflectance in the near infrared waveband explained 84, 88, 79, 90 and 85% of the variance, respectively, in these forage quality variables. Generally, the correlations of forage quality parameters with the first derivatives of reflectance were much higher than r^2^ of the reflectance (Table 4). Recently, researchers have evaluated remote sensing techniques for estimating the nutrient status of growing crops by determining the appropriate wavelength or combination of wavelengths to characterize crop nutrient deficiency [3].

Reflectance measured with hand-held radiometers has been used to estimate many plant parameters of interest [2]. It is stated that crude protein or N has been correlated with spectra using handheld sensors for grasses [8, 21]. Blackmer *et al.* [29] reported that reflected radiation near 550 and 710 nm was better for detecting N deficiencies compared with reflectance at other wavelengths. Stone *et al.* [26] demonstrated that total plan N could be estimated using spectral radiance measurements at the NIR (780 nm) wavelengths. This result is consistent with the present results for NIR waveband.

Başayiğit and Albayrak [30] found that the r^2^ of predicted and measured N, P and K were high (0.94, 0.80 and 0.88, respectively) in wollypod vetch. They concluded that spectral reflectance in VNIR can be used for nondestructive prediction of forage N, P and K content in wollypod vetch. This result is consistent with our results.

The four most important wavebands for each measured forage quality variable and the corresponding multivariable equations and r^2^ values, obtained by stepwise regression, are given in Table 5. The stepwise regression of reflectance explained 85, 85, 78, 81 and 74% of the variance, respectively, in forage N, P, K, ADF and NDF. The stepwise regression of the first derivatives of reflectance explained 87, 91, 83, 93 and 86% of the variance, respectively, in these forage quality variables. Generally, the correlations of forage quality parameters with the stepwise regression of the first derivatives of reflectance were much higher than r^2^ of stepwise regression of reflectance (Table 5). Compared with the linear and stepwise regression analyses, the stepwise relationships between forage N, P, K, ADF and NDF concentration and canopy reflectance were much higher than r^2^ of linear regression (Table 4, 5).

The common method for acquiring data from multi-spectral or hyperspectral systems is that they were converted and averaged into reflectance of blue, green, red, near-infrared (450-520, 520-600, 630-690, 760-900 nm) wavebands [31, 32]. The reflectance of the broad wavebands was then used for predicting plant parameters such as leaf N concentration, leaf chlorophyll concentration, leaf area index, grain yield using simple ratio or normalized vegetation index [33-35]. Present study, broad wavebands had high correlated with forage quality variables. Stepwise regression has been widely used to relate remotely sensed data to vegetation variables [36-39]. The selection of wavelengths by stepwise regression is an important step towards the development of general models for predicting chemicals in sainfoin pasture. Several publications have shown a strong relationship between the concentration of nitrogen and concentrations of chlorophyll a and b [7, 8, 40]. Nitrogen is related to the protein synthesis that promotes the photosynthetic process. Therefore, nitrogen deficiency disturbs the metabolic function of the chlorophyll, which is the photosynthetic element responsible for the absorption of electromagnetic energy at specific wavelengths in the visible region [7, 8].

Since chlorophyll largely determines spectral reflectance in the visible, a strong relationship between visible absorption bands and nitrogen concentration is also expected. The same applies to other biochemicals such as phosphorous and potassium which are also responsible for both the photosynthetic process and tissue composition in plants [8]. Starks *et al.* [21] reported that forage CP high correlated with broad waveband in stepwise regression analysis. Starks *et al.* [13] analyzed the relationships between canopy reflectance in 252 wavebands, covering the 368 to 1100 nm region, and forage quality variables (i.e. NDF, ADF, and N concentrations) of bermudagrass pasture using stepwise regression methods. They found that forage N, NDF, and ADF concentrations closely and linearly correlated with pasture canopy reflectance. This result is consistent with the present results.

First derivatives of reflectance in visible near infrared region (400-900 nm) have been used to estimate leaf N concentration in ryegrass [12] and in sorghum [41]. Similar to previous reports, both the reflectance and the first derivatives of reflectance (Figure 3) in most wavebands in the present study improved linear relationships between canopy reflectance and N, P, K, ADF and NDF availability. In present study, the wavelengths selected and used for linear and stepwise regression equations were close to those reported previously by other researchers [8, 12, 13, 26, 28-30, 41].

## 4. Conclusions

The simple ratio of reflectance in near infrared (NIR) to red regions (R(NIR)/R(red)) of the electromagnetic spectrum and the normalized difference vegetation index (NDVI), defined as (R(NIR) - R(red))/(R(NIR) + R(red)), were highly with nitrogen, phosphorus, potassium, acid detergent fiber and neutral detergent fiber contents of sainfoin pasture. Forage quality variables closely correlated with canopy reflectance of near infrared waveband (780 nm). These five forage variables were also correlated with the first derivatives of reflectance in near infrared waveband (760 nm). All forage quality variables were higher correlated with the stepwise regression of reflectance (460, 550, 650 and 780 nm) and stepwise regression of the first derivatives of the reflectance (440, 530, 630 and 760 nm). The results of validating the developed linear and stepwise models indicated that relationships of forage N, P, K, ADF and NDF contents with sainfoin pasture canopy reflectance were high. The quality parameters could be adequately predicted using reflectance or first derivatives of reflectance. Therefore, canopy reflectance in blue, green, red and near infrared wavebands with NIR/Red and NDVI ratios can be used for nondestructive assessment of forage quality variables in sainfoin pasture.

## Figures and Tables

**Table 1.** N, P, K, ADF and NDF contents of sainfoin pasture (n=50).

**Parameter**	**N**	**P**	**K**	**ADF**	**NDF**

**Maximum**	3.54	0.94	2.88	37.76	44.21
**Minimum**	2.15	0.16	1.03	27.54	35.43
**Mean**	2.74	0.50	1.66	32.46	39.53
**SD**	0.40	0.22	0.52	2.60	2.20
**CV (%)**	14.64	44.21	31.05	8.02	5.56

**Table 2.** Relationships between NIR/RED ratio and nutrition contents in sainfoin pasture.

**Quality parameter**	**Equation**	**Standard error**	**^2^**

NIR/RED			

N (%)	N = 2.19+0.07×(R780/650)	0.209	0.73***
P (%)	P = 0.21+0.04×(R780/650)	0.125	0.69***
K (%)	K = 0.93+0.09×(R780/650)	0.232	0.80***
ADF (%)	ADF = 35.66-0.39×(R780/650)	1.989	0.61***
NDF (%)	NDF = 42.24-0.33×(R780/650)	1.387	0.61***

First derivatives of NIR/RED			

N (%)	N = 1.41+0.002×(R760/630)	0.172	0.82***
P (%)	P = -0.23+0.001×(R760/630)	0.099	0.80***
K (%)	K = -0.07+0.003×(R760/630)	0.208	0.84***
ADF (%)	ADF = 40.76-0.01×(R760/630)	1.298	0.76***
NDF (%)	NDF = 46.28-0.01×(R760/630)	1.210	0.70***

Probability level, P<0.0001

**Table 3.** Relationships between NDVI and nutrition contents in sainfoin pasture.

**Quality parameter**	**Equation**	**Standard error**	**r^2^**

NDVI			

N (%)	N = 0.83+2.66×(R NDVI)	0.183	0.80***
P (%)	P = -0.54+1.46×(R NDVI)	0.105	0.78***
K (%)	K = -0.69+3.27×(R NDVI)	0.270	0.73***
ADF (%)	ADF = 44.19-16.35×(R NDVI)	1.654	0.72***
NDF (%)	NDF = 49.50-13.88×(R NDVI)	1.165	0.72***

First derivatives of NDVI			

N (%)	N = -350.89+355.01×(R NDVI)	0.173	0.82***
P (%)	P = -195.3+196.56×(R NDVI)	0.095	0.82***
K (%)	K = -436.89+440.25×(R NDVI)	0.253	0.76***
ADF (%)	ADF = 2291.77-2268.08×(R NDVI)	1.196	0.79***
NDF (%)	NDF = 1914.88-1882.63×(R NDVI)	1.069	0.77***

Probability level, P<0.0001

**Table 4.** Near infrared wavebands (±5) selected from linear regression for determining relationships between reflectance and nutrition contents on the basis of calibration data set in sainfoin pasture.

**Quality parameter**	**Equation**	**Standard error**	**r^2^**

Reflectance			

N (%)	N = 0.54+4.24×(R780)	0.168	0.83***
P (%)	P = -0.70+2.32×(R780)	0.097	0.81***
K (%)	K = -1.02+5.17×(R780)	0.262	0.75***
ADF (%)	ADF = 46.38-26.85×(R780)	1.209	0.79***
NDF (%)	NDF = 50.46-21.08×(R780)	1.249	0.68***

First derivatives of reflectance			

N (%)	N = 0.02+402.76×(R760)	0.164	0.84***
P (%)	P = -1.04+228.56×(R760)	0.077	0.88***
K (%)	K = -1.73+500.97×(R760)	0.241	0.79***
ADF (%)	ADF = 50.82-2716.2×(R760)	0.812	0.90***
NDF (%)	NDF = 54.58-2226.52×(R760)	0.848	0.85***

Probability level, P<0.0001

**Table 5.** Four wavebands (±5) selected from stepwise regression for determining relationships between reflectance and nutrition contents on the basis of calibration data set in sainfoin pasture.

**Quality parameter**	**Equation**	**SE**	**r^2^**

Stepwise regression of reflectance			

N (%)	N = 1.61-2.20×(R460)+0.55×(R550)-2.47×(R650)+2.79×(R780)	0.163	0.85***
P (%)	P = 0.20-2.28×(R460)-0.73×(R550)-0.53×(R650)+1.18×(R780)	0.090	0.85***
K (%)	K = 1.09-2.79×(R460)-1.17×(R550)-4.28×(R650)+2.45×(R780)	0.251	0.78***
ADF (%)	ADF=42+31.6×(R460)-10.7×(R550)-4.04×(R650)-0.21×(R780)	1.182	0.81***
NDF (%)	NDF=40.7+23.8×(R460)-8.7×(R550)+22×(R650)-7.6×(R780)	1.169	0.74***

Stepwise regression of the first derivatives of reflectance			

N (%)	N=1.83-1372.5×(R440)-125×(R530)-2136×(R630)+271×(R760)	0.151	0.87***
P (%)	P=0.15-3279×(R440)-100.6×(R530)-2080×(R630)+143×(R760)	0.069	0.91***
K (%)	K=1.02-4097×(R440)+68.4×(R530)-1366×(R630)+326×(R760)	0.219	0.83***
ADF (%)	ADF=40+42531×(R440)+1517×(R530)-229119×(R630)-1932×(R760)	0.703	0.93***
NDF (%)	NDF=51+26981×(R440)-341×(R530)-22446×(R630)-2020×(R760)	0.859	0.86***

SE: Standard error, Probability level, P<0.0001
